# Efficacy and Safety of Bilateral Deep Brain Stimulation (DBS) for Severe Alzheimer's Disease: A Comparative Analysis of Fornix Versus Basal Ganglia of Meynert

**DOI:** 10.1111/cns.70285

**Published:** 2025-04-17

**Authors:** Junpeng Xu, Bin Liu, Guosong Shang, Zhebin Feng, Haonan Yang, Yuhan Chen, Xinguang Yu, Zhiqi Mao

**Affiliations:** ^1^ Medical School of Chinese PLA Beijing China; ^2^ Department of Neurosurgery The First Medical Center of Chinese PLA General Hospital Beijing China; ^3^ PLA 942 Hospital Yinchuan China; ^4^ The First Clinical Medical College of Hebei North University Zhangjiakou China

**Keywords:** deep brain stimulation, fornix, nucleus basalis of Meynert, severe Alzheimer's disease

## Abstract

**Background:**

Deep brain stimulation (DBS) is a novel therapy for severe Alzheimer's disease (AD). However, there is an ongoing debate regarding the optimal target for DBS, particularly the fornix and the basal ganglia of Meynert (NBM).

**Objective:**

This study aimed to investigate the safety and efficacy of DBS for severe AD and to compare the fornix and the NBM as potential targets.

**Methods:**

We conducted a prospective, nonrandomized clinical study involving 20 patients with severe AD (MMSE score 0 to 10, CDR level 3) from January 2015 to August 2022, comprising 12 males and eight females, with a mean age of 59.05 ± 6.45 years. All patients underwent DBS treatment, among which 14 received bilateral fornix implantation, while six received bilateral implantation in the NBM. Electrical stimulation commenced 1 month postoperatively. We assessed the patients before surgery, followed by evaluations at 1 month, 3 months, 6 months, and 12 months poststimulation. Primary outcome measures focused on changes in cognitive function, assessed using the MMSE, MoCA, ADAS‐Cog, and CDR scales. Secondary measures encompassed quality of life, caregiver burden, neuropsychiatric symptoms, and sleep disturbances, evaluated through the BI, FAQ, FIM, ZBI, NPI, HAMA, HAMD, and PSQI scales.

**Results:**

All patients tolerated DBS well, with no serious adverse effects reported. Early on, DBS significantly improved cognitive function and quality of life. Long‐term benefits include the improvement of neuropsychiatric symptoms and sleep disorders and the alleviation of caregiver burden. Comparison between DBS targeting the NBM and fornix revealed no significant differences in overall scale scores. However, upon deeper analysis, NBM‐DBS exhibited a more pronounced improvement in neuropsychiatric symptoms, particularly in NPI scores.

**Conclusion:**

DBS is a potential therapeutic approach for severe AD, capable of improving patients' cognitive function, quality of life, and neuropsychiatric symptoms. Notably, NBM‐DBS showed distinct advantages in ameliorating neuropsychiatric symptoms, providing valuable insights for clinically selecting the optimal DBS target.

**Trial Registration:**

ClinicalTrials.gov identifier: NCT03115814

## Graphical abstract

The graphical abstract illustrates the key findings of a study comparing fornix‐DBS and NBM‐DBS in severe Alzheimer’s disease. It highlights the localization of the fornix (Figure A) and the NBM (Figure C), and the structure of the DBS device (Figure B). The main results are summarized in Figure D, showing that both fornix‐DBS and NBM‐DBS are effective in improving cognitive function and quality of life, but NBM‐DBS demonstrates a more significant improvement in neuropsychiatric symptoms, particularly in NPI scores. Overall, DBS shows beneficial effects but does not significantly alter the clinical prognosis of patients.

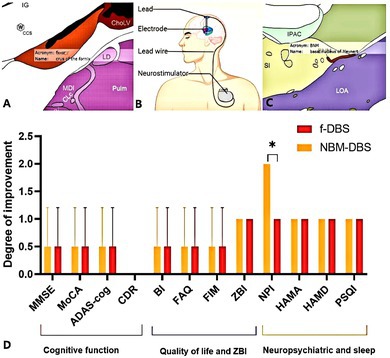

## 1 Introduction

The number of dementia patients will reach 200 million in the middle of this century [1]. Alzheimer's disease (AD) comprises approximately 75% of all dementia cases and represents the most prevalent form of dementia [2, 3]. Apart from severe cognitive dysfunction, approximately 80% of patients with severe AD also exhibit neuropsychiatric symptoms (NPS), including delusions, hallucinations, depression, anxiety, apathy, and mania [4, 5]. These NPS symptoms lead to increased rates of misdiagnosis and missed diagnosis. Murray et al.'s research revealed that 53% of AD patients presenting with NPS symptoms were misdiagnosed as having mental illnesses, whereas the misdiagnosis rate for those without NPS symptoms was only 4% [6]. Delayed diagnosis resulting from misdiagnosis and missed diagnosis robs patients of the optimal timing for early intervention, thereby increasing the mortality rates and caregiver burden associated with AD [7]. Currently, there is no definitively effective treatment for severe AD and virtually all AD patients will inevitably progress to the severe stage.

Traditional drug therapies are degraded by limited efficacy, high cost, and significant side effects. A survey reported on AD drug treatment indicated that 70.66% of patients spent over 40,000 yuan annually on drug treatment for the disease, 63.79% of patients believed therapy was ineffective, and 64.87% found the drug prices to be excessively high [8]. Additionally, 34.74% of patients reported significant side effects, and the proportion of patients who discontinued medication due to these issues reached 43.49%. Existing research confirms that current drug treatments are primarily suitable for patients with mild to moderate AD but do not improve patient prognosis. Novel monoclonal antibodies, such as Bapineuzumab, Gantenerumab, and Lecanemab, reduce amyloid‐beta (Aβ) protein deposition in the brain but fail to stop AD progression or significantly improve cognitive function [9, 10, 11, 12]. Similarly, Tau aggregation inhibitors like TRx0237 and Gosuranemab have not shown significant efficacy in clinical trials for mild AD patients. AADvac1, a vaccine targeting pathological Tau protein clearance, exhibits promising safety and efficacy profiles but requires large‐scale trials for further validation [13]. Neuroinflammation inhibitors, including Daratumumab, NE3107, and Masitinib, have shown cognitive improvements in mild AD patients in preliminary studies but also require broader validation [14]. As AD progresses to severe stages, pharmacological treatments become less effective, patients suffer from physiological dysfunction and neuropsychiatric symptoms, and the societal and economic burden increases. Multidrug therapies are often necessary but can result in drug interactions and increased side effects, complicating care for patients with compromised liver and kidney functions.

Beyond pharmacological interventions, nonpharmacological approaches such as noninvasive brain stimulation (NIBS), including repetitive transcranial magnetic stimulation (rTMS) [15, 16] and transcranial direct current stimulation (tDCS) [17, 18, 19], are promising due to their high safety profiles and precise targeting capabilities. Transcranial alternating current stimulation (tACS) [20, 21, 22, 23], which has demonstrated cognitive enhancement in healthy individuals and patients with mild cognitive impairment (MCI), has been less studied in AD patients, with existing research primarily focusing on short‐term effects and yielding unsatisfactory long‐term follow‐up results. Cognitive rehabilitation training, another nonpharmacological approach, requires professional guidance to ensure safety and efficacy. However, this therapy is resource‐intensive and necessitates long‐term periodic sessions, making it unfeasible for many families.

**FIGURE 1:**
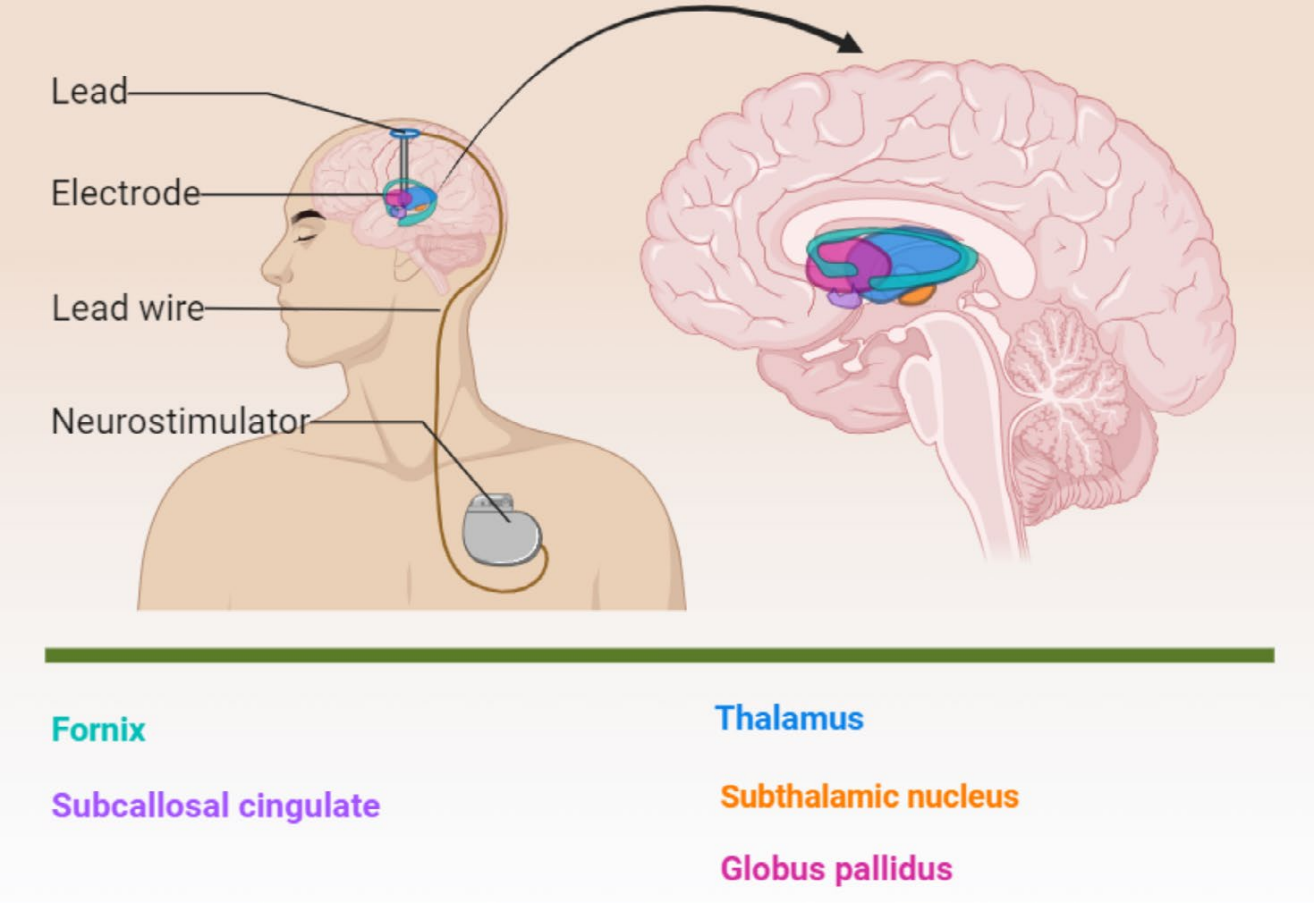
Schematic diagram of DBS for the treatment of severe AD.

The prognosis for severe AD patients remains poor, and current treatment modalities have significant limitations in improving long‐term outcomes. While the above treatment methods show some progress in improving cognitive function in AD patients, their long‐term effects are uncertain. The limited efficacy in AD patients may stem from the unclear pathogenesis of the disease. Recent studies have revealed neuronal cell death, atrophy, and degradation in the nucleus basalis of Meynert (NBM) and the fornix among AD [24, 25]. Segtnan et al. [26] introduced the concept of neural circuit disorders, suggesting that AD disrupts or alters multiple cortical and subcortical connections vital for cognitive and memory functions. Regulating neuronal and associated network activities thus emerges as a potential therapeutic approach [27]. Given this backdrop, researchers have explored the use of DBS for AD. DBS in AD is also primarily focused on single‐target stimulation, and three primary targets for AD patients are the NBM [27, 28], the fornix [29], and the ventral capsule/ventral striatum (VC/VS) region [30]. There is considerable debate regarding the optimal target, one of which lies in the choice between stimulating the fornix and the NBM (Figure 1). Both targets have shown promise in preliminary studies, but the consensus on which is more effective has not yet been reached. Several clinical studies have thoroughly investigated the potential therapeutic benefits of fornix‐DBS and NBM‐DBS in managing memory loss and cognitive decline in AD. Kuhn et al. (2015) reported significant cognitive improvement in 4 out of 6 mild‐to‐moderate AD patients treated with DBS‐NBM. Previous clinical trials of NBM‐DBS have predominantly focused on alleviating cognitive symptoms in mild to moderate AD [31, 32]. Mao et al. (2018) focused on advanced AD patients, observing a 25% average improvement in global cognitive function tests within 1.5 to 3 months of fornix‐DBS [33]. In 2022, Zhang et al. further reported on eight advanced AD patients who underwent NBM‐DBS, revealing short‐term cognitive enhancement but no significant long‐term changes at the 12‐month follow‐up [34].

These studies have underscored the potential of DBS in mitigating cognitive decline in AD, with a particular focus on expanding the scope from mild to moderate stages, as demonstrated in the work by Zhang et al. However, these studies often undervalue the significant impact of neuropsychiatric symptoms, sleep disorders, and the resulting decline in quality of life and psychological stress on patients and their families. Recognizing the need to address these noncognitive aspects of AD, which are equally debilitating, we aim to develop a comprehensive treatment plan for AD symptoms, including neuropsychiatric and behavioral abnormalities and sleep disorders, along with their effects on patients' quality of life and caregivers' well‐being. To this end, our study, involving 20 severe AD patients who underwent DBS surgery, seeks to explore the safety and efficacy of DBS for severe AD, compare the benefits of fornix and NBM stimulation, and provide theoretical and technical insights to guide treatment strategies.

## 2 Methods

### 2.1 Patient Enrollment and Informed Consent

**FIGURE 2:**
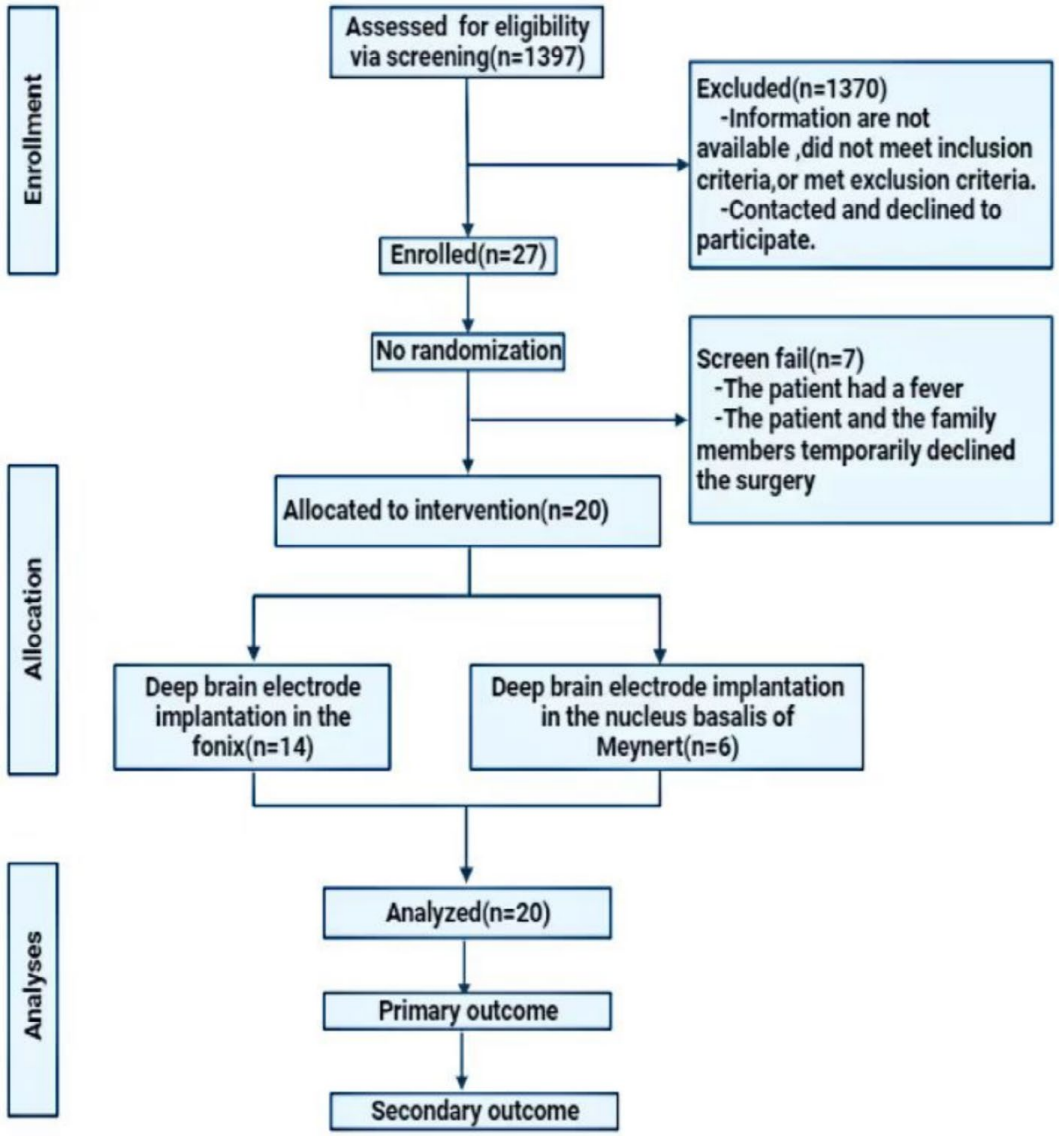
Flowchart of study participants.

From 1397 patients diagnosed with AD, we screened and enrolled 27 individuals who met our inclusion and exclusion criteria at the PLA Hospital from January 2015 to August 2022. We excluded seven participants due to surgical contraindications or the inability to obtain valid informed consent from patients or their families, and our final study cohort comprised 20 patients with severe AD (Figure 2). In our study, we included patients aged 40 to 80 with AD diagnosed according to NINCDS‐ADRDA criteria, a CDR of 3.0, and MMSE and MoCA scores between 0 and 10 who provided informed consent and were willing to cooperate with the trial. We excluded those with preoperative brain abnormalities, other neurological disorders, severe medical conditions, a history of cranial surgery, contraindications for MRI or tDCS, skin issues, other dementia types, and any other conditions deemed unsuitable by the investigator. Patients could withdraw if they experienced severe side effects, refused treatment, or were lost to follow‐up (Table 1). Recognizing the intricacies and ethical considerations involved in conducting a DBS study in AD patients with profound cognitive impairments, we prioritized the informed consent process with utmost care and sensitivity. We ensured that those authorized to provide consent did so following ethical standards. To assist patients in understanding, we utilized communication methods aligned with their cognitive abilities, providing repeated explanations as necessary. In special cases, we obtained informed consent through legal representatives and communication aids to respect their autonomy. We maintained an ongoing dialogue with patients and their families to establish trust and understanding. Our procedures adhered to the guidelines of our ethics committee, ensuring the ethical integrity of the study and protecting all participants.

**TABLE 1:** List of inclusion and exclusion criteria.

Inclusion	Exclusion
Age 40–80 years, male or female Meeting the diagnosis criteria of AD formulated by the National Institute of Neurological and Communicative Disorders and Stroke and the Alzheimer's Disease and Related Disorders Association (NINCDS‐ADRDA) Clinical Dementia Rating Scale (CDR) =3.0 MMSE score (0–10) MoCA score (0–10) Provision of signed informed consent The patient or his guardian agrees to participate in this trial and is able to cooperate with the follow‐up work	The presence of preoperative structural brain abnormalities (such as tumors, cerebral infarction, hydrocephalus, or intracranial hemorrhage) The presence of other neurological disorders such as multiple sclerosis, epilepsy, and Parkinson's disease Severe medical illness, current use of respiratory medications, cardiovascular medications, anticonvulsants or psychoactive drugs, and clinically significant gastrointestinal, renal, hepatic, respiratory, infectious, endocrine or cardiovascular disease, cancer, alcoholism, or drug addiction Patients who have undergone cranial surgery Contraindications to undergoing magnetic resonance imaging or receiving transcranial alternating current stimulation (pacemakers, postdeep brain stimulation (DBS) surgery) Eczema or sensitive skin Presence of other types of dementia: vascular dementia, Lewy body dementia, frontotemporal dementia, infectious dementia, etc. Other conditions that, in the opinion of the investigator, may not be suitable for this study
Withdrawal criteria	
Serious side effects occur, causing safety problems for the patient Patients refuse to continue treatment Loss to follow‐up	
Termination criteria	
Continuation of the study may harm the relevant rights and interests of a certain number of subjects	

### 2.2 Intervention

#### 2.2.1 Bilateral DBS Implantation

**FIGURE 3:**
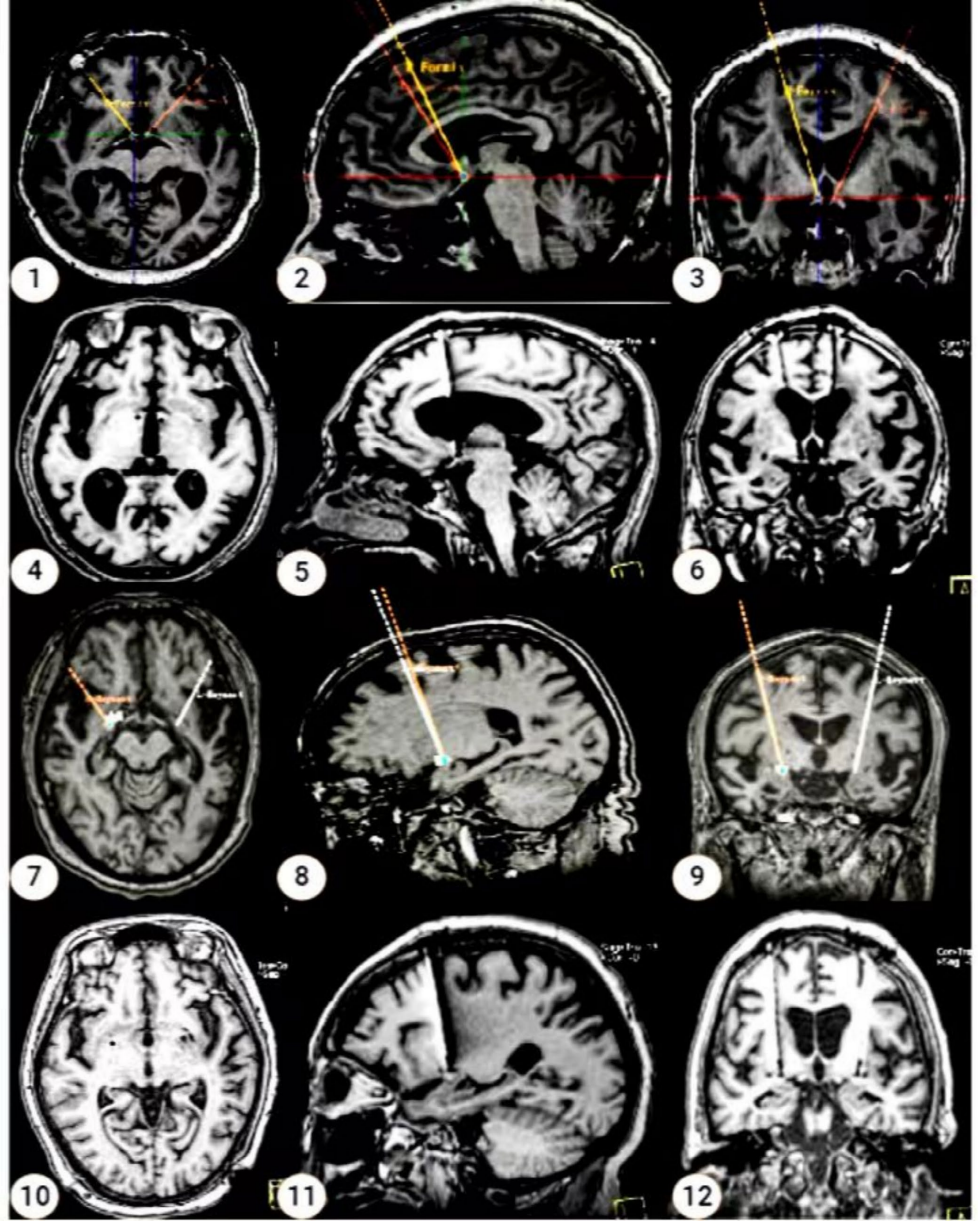
Preoperative planning and postoperative electrode position diagram of DBS. 1–6: Preoperative planning and postoperative electrode positions of bilateral fornix‐DBS; 7–12: Preoperative planning and postoperative electrode positions of bilateral NBM‐DBS.

Under general anesthesia, the patient was fitted with a device and underwent intraoperative MRI scans to ensure precise localization of the NBM and fornix. We utilized microelectrodes to record electrophysiological signals, which helped confirm the anatomical position and allowed us to select the most active electrode channel. DBS leads (L301C; Beijing PINS Medical) were then implanted at the targeted site, and intraoperative MRI confirmed that the electrode placement was satisfactory (Figure 3).

#### 2.2.2 Programmed Control Method

For severe AD, we implemented personalized DBS protocols tailored to each individual's needs. Beginning 4 weeks postoperatively, we utilized a unipolar stimulation mode, initiating stimulation at the fornix at 130 Hz with a pulse width of 90 μs and at the NBM at 20 Hz with a pulse width of 90 μs. The initial voltage was gradually increased from 0.5 to 1 V to achieve a therapeutic voltage within the range of 2–5 V. Throughout the initial stimulation phase, patients were monitored for 30 min to assess any behavioral changes, improvements in communication, and alleviation of symptoms. Following this, a neuropsychological assessment was conducted to track cognitive enhancements and to guide adjustments for optimal DBS efficacy. We selected the contacts and parameters for chronic stimulation. Patients were followed up at 3, 6, 9, and 12 months postsurgery to reassess their functional capabilities and to adjust the stimulation parameters as needed, which monitored treatment outcomes, ensured patient safety, and provided tailored care. Postoperatively, patients commenced electrical stimulation, specifically targeting NBM‐DBS and bilateral fornix‐DBS, minimizing potential adverse effects while maximizing the therapeutic benefits of the intervention.

### 2.3 Data Collection

**FIGURE 4:**
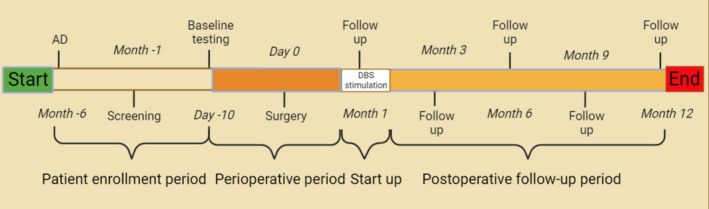
Time stages of clinical trials.

Third‐party researchers and research assistants collected data and followed up through outpatient face‐to‐face interactions. We collected baseline information before surgery and conducted follow‐ups at 3, 6, 9, and 12 months after surgery (Figure 4). At each time point, we used standardized and structured questionnaires to gather data, with the primary outcome being the change in cognitive function before and after treatment. Cognition is the core domain in the assessment of AD, and we evaluated patients' cognitive function through the Mini‐Mental State Examination (MMSE), Montreal Cognitive Assessment (MoCA), Alzheimer's Disease Assessment Scale‐Cognitive Subscale (ADAS‐Cog), and Clinical Dementia Rating Scale (CDR). Quality of life is a key indicator of an AD patient's overall health status, and we utilized the Barthel Index (BI), Functional Activities Questionnaire (FAQ), and Functional Independence Measure (FIM) to assess patients' quality of life. Neuropsychiatric symptoms and sleep disturbances are common complications in AD patients, affecting their daily functioning, social interactions, and family relationships. We assessed neuropsychiatric symptoms through the Neuropsychiatric Inventory (NPI), Hamilton Anxiety Rating Scale (HAMA), Hamilton Depression Rating Scale (HAMD), and Pittsburgh Sleep Quality Index (PSQI). Furthermore, AD not only affects the patients themselves but also imposes psychological and financial burdens on caregivers, making it a significant assessment indicator. Therefore, we chose the Zarit Burden Interview (ZBI) to evaluate caregiver burden and provide necessary support and interventions.

### 2.4 Statistical Analysis

Two independent and unbiased third‐party administrators conducted rigorous data analysis using SPSS 25.0. Throughout the statistical analysis, we adhered strictly to scientific principles and performed comprehensive normality assessments on the entire dataset to ascertain its conformity to a normal distribution. For data adhering to a normal/Gaussian distribution, we employed statistical methodologies for such distributions, thereby enhancing the precision and credibility of our findings. Conversely, for data that did not meet the criteria for normality, we diligently utilized nonparametric alternative methods like the Mann–Whitney U test or Wilcoxon signed‐rank test to analyze the non‐normal distributions and derive robust statistical conclusions. The categorical variables in our study are presented as case counts and percentages, allowing for a clear and concise overview of the distribution of the variables among the study population. The statistical comparisons were conducted using either the chi‐squared test or Fisher's exact test, depending on the appropriateness of each method for the given data. For continuous variables, we presented them as mean ± standard deviation (SD) or median (with the range indicated). When analyzing normally distributed continuous variables, we relied on paired *t*‐tests. To assess changes in scale scores over time, we utilized repeated measures ANOVA. When analyzing normally distributed continuous variables, we employed paired t‐tests. To evaluate changes in scale scores across time points, we utilized repeated measures ANOVA. For data that did not adhere to normal distribution, we opted for the Wilcoxon signed‐rank test and generalized estimating equations (GEEs) to ensure rigorous and accurate statistical analysis. To mitigate the risk of Type I error and uphold the rigor of the Bonferroni correction method, given the conventional significance level of α = 0.05, we adjusted the significance threshold for each hypothesis test to α / 2 = 0.025. This adjustment strengthened the statistical inferences we could draw from our analyses. A two‐sided *p* value of less than 0.05 indicated statistically significant differences between samples.

## 3 Results

### 3.1 Patients

We included 20 patients with AD (MMSE scores of 0–10, CDR level 3) admitted to the PLA Hospital from January 2015 to August 2022, including 11 males and nine females (Figure 2). The average age is 59.05 ± 6.45 years, the average education years are 10.40 ± 3.30 years, the average disease duration is 5.60 ± 2.35 years, the average BMI is 22.82 ± 3.68Kg/m2, the average operation time is 5.92 ± 1.82 h, and the average hospitalization length is 30.70 ± 16.69 days. The average number of hospitalizations was 1.40 ± 0.60, and the average follow‐up time was 59.70 ± 28.41 months. Notably, all patients (100%) exhibited severe memory loss, and 65% presented with neuropsychiatric symptoms such as impulsivity, aggression, depression, and emotional indifference. Additionally, 30% had reduced calculation abilities, 15% showed slow reactions and mental retardation, and 25% presented with psychiatric disorders. All patients underwent DBS treatment, with 14 receiving bilateral fornix implants and six receiving NBM implants (Table 1). The fornix‐DBS group had an average age of 59.86 years, 10.21 years of education, and a disease duration of 6.00 years. They had a lower average BMI of 21.63 kg/m^2, an average operation time of 6.14 h, and an average hospital stay of 29.64 days. Their average follow‐up time was significantly longer at 75.36 months compared to the NBM‐DBS group. The NBM‐DBS group had an average age of 57.17 years, 10.83 years of education, and a shorter disease duration of 4.67 years. Their average BMI was higher, with an average operation time of 5.42 h, a hospital stay of 33.17 days, and an average follow‐up time of 23.17 months, shorter than the fornix‐DBS group. Statistical analysis revealed that the fornix‐DBS group had a longer follow‐up time and lower BMI than the NBM‐DBS group. No significant differences were found in other baseline characteristics, indicating that both patient groups were similar at the study's onset, ensuring comparable results (Table 2).

**TABLE 2:** Baseline characteristics of study participants.

Characteristic	Total	f‐DBS	NBM‐DBS	*p*
Number	20	14	6	
Age (years) mean ± SD	59.05 ± 6.45	59.86 ± 6.20	57.17 ± 7.22	0.407
Gender				0.050
Male	11	10	1	
Female	9	4	5	
Treatment of AD drugs				NA
Yes	20	14	6	
No	0	0	0	
Other neuroregulatory treatments				1.000
Yes	3	2	1	
No	17	12	5	
Family history				0.202
Yes	3	1	2	
No	17	13	4	
Accompanied by neuropsychiatric symptoms				0.354
Yes	13	8	5	
No	7	6	1	
Education	10.40 ± 3.30	10.21 ± 3.38	10.83 ± 3.37	0.711
Course of disease	5.60 ± 2.35	6.00 ± 2.25	4.67 ± 2.50	0.255
BMI	22.82 ± 3.68	21.63 ± 2.44	25.60 ± 4.75	0.113
Cognitive assessments				
MMSE	5.20 ± 3.04	5.29 ± 3.41	5.00 ± 2.19	0.738
MoCA	4.25 ± 2.79	4.21 ± 3.17	4.33 ± 1.86	0.900
ADAS‐cog	51.05 ± 11.39	50.86 ± 12.13	51.50 ± 10.50	0.803
CDR	3.00 ± 0.00	3.00 ± 0.00	3.00 ± 0.00	NA
Psychiatric symptoms assessment				
NPI	26.90 ± 4.17	26.07 ± 3.89	28.83 ± 4.49	0.181
HAMA	16.30 ± 5.35	16.07 ± 5.58	16.83 ± 5.23	0.779
HAMD	20.70 ± 5.35	20.57 ± 5.72	21.00 ± 4.86	0.934
PSQI	14.40 ± 1.43	14.14 ± 1.51	15.00 ± 1.10	0.330
Quality of life assessment				
Barthel Index	60.75 ± 17.94	61.43 ± 19.26	59.17 ± 15.94	0.804
FAQ	26.80 ± 3.00	26.36 ± 3.43	27.83 ± 1.33	0.224
FIM	71.50 ± 22.65	73.00 ± 24.22	68.00 ± 20.07	0.680
ZBI	50.55 ± 10.40	50.07 ± 11.82	51.67 ± 6.74	0.763

### 3.2 Effects on Cognitive Function

**FIGURE 5:**
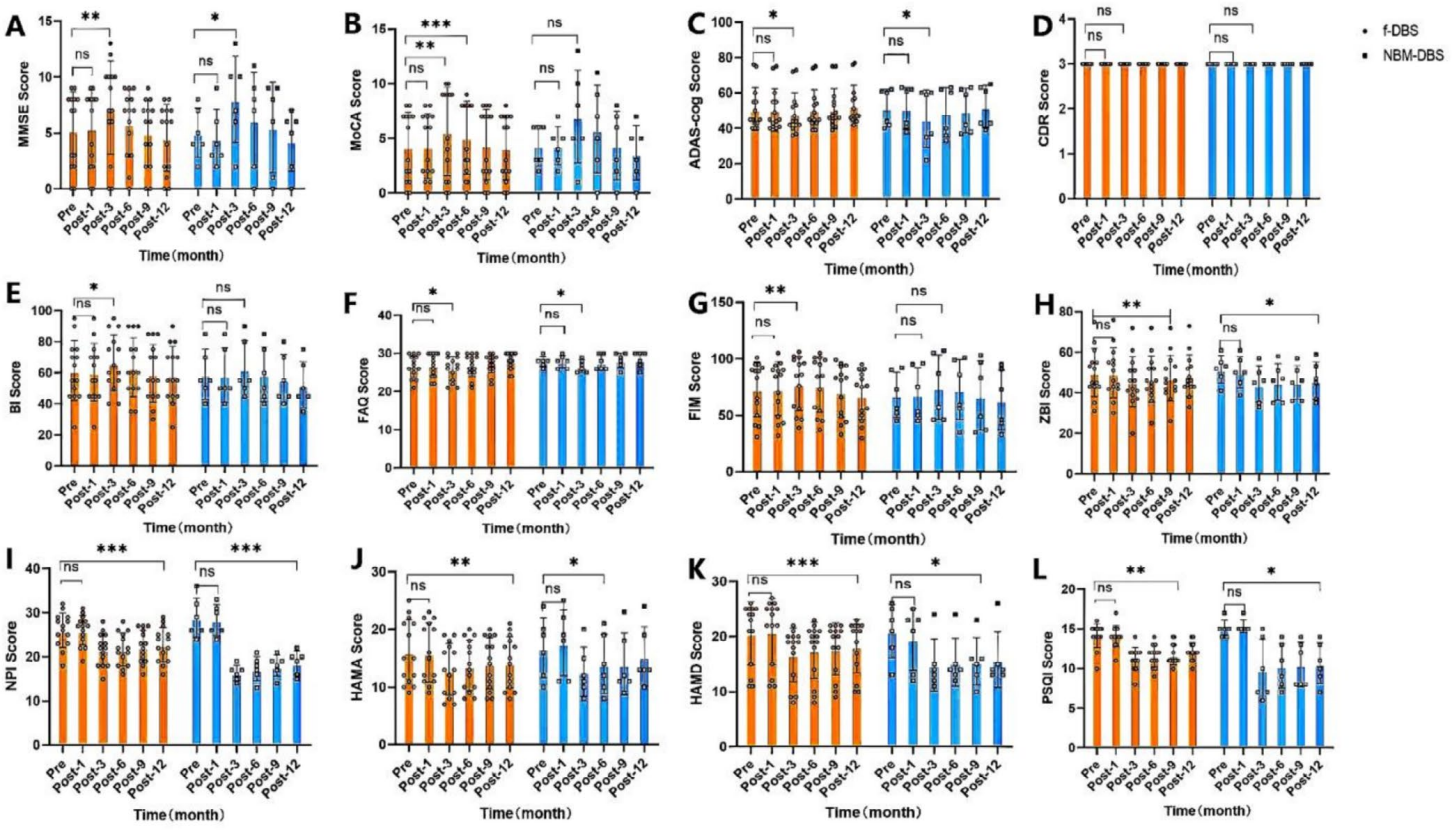
Overall patient score changes in different time periods.

Baseline MMSE scores were 5.20 ± 3.04 and CDR grade 3. At 3 months, MMSE significantly improved to (7.50 ± 3.98, *p* < 0.05). MoCA scores followed a similar pattern, significantly improving at 3 months (6.05 ± 4.03, *p* < 0.05). ADAS‐cog scores showed a significant downward trend (47.60 ± 12.84, *p* < 0.05). However, this improvement was not sustained, with scores at 6, 9, and 12 months not different from the baseline. The MMSE, MoCA, ADAS‐cog, and CDR scale scores at each time point were compared between the NBM‐DBS group and the fornix‐DBS group, with none indicating significant differences (*p* > 0.05) (Figure 5, Tables 3, 4, 5).

**TABLE 3:** Primary outcome at 1, 3, 6, 9, and 12 months.

Scales	Baseline	1 month		3 month		6 month		9 month		12 month	
	Value	Value	*p*	Value	*p*	Value	*p*	Value	*p*	Value	*p*
Cognitive assessments											
MMSE	5.20 ± 3.04	5.15 ± 3.15	0.796	7.50 ± 3.98	0.001	5.95 ± 3.32	0.098	5.15 ± 3.25	0.765	4.50 ± 2.84	0.028
NBM‐DBS	5.00 ± 2.19	4.50 ± 2.59	0.203	8.00 ± 3.85	0.037	6.17 ± 4.26	0.443	5.50 ± 4.04	0.733	4.33 ± 2.73	0.530
f‐DBS	5.29 ± 3.41	5.43 ± 3.41	0.527	7.29 ± 4.16	0.004	5.86 ± 3.01	0.054	5.00 ± 3.01	0.206	4.57 ± 2.98	0.013
MoCA	4.25 ± 2.79	4.30 ± 2.60	0.763	6.05 ± 4.03	0.001	5.30 ± 3.47	0.004	4.40 ± 3.12	0.359	4.00 ± 2.77	0.334
NBM‐DBS	4.33 ± 1.86	4.33 ± 1.75	1.000	7.00 ± 4.24	0.072	5.83 ± 4.02	0.216	4.33 ± 3.14	0.891	3.67 ± 2.50	0.317
f‐DBS	4.21 ± 3.17	4.29 ± 2.95	0.655	5.64 ± 4.03	0.004	5.07 ± 3.34	0.001	4.43 ± 3.23	0.180	4.14 ± 2.96	0.739
ADAS‐cog	51.05 ± 11.39	50.75 ± 11.58	0.153	47.60 ± 12.84	0.001	50.05 ± 11.93	0.145	50.85 ± 11.39	0.667	52.75 ± 11.14	0.005
NBM‐DBS	51.50 ± 10.50	51.33 ± 11.81	0.739	45.50 ± 16.36	0.027	49.00 ± 14.09	0.223	50.17 ± 12.42	0.336	52.33 ± 11.84	0.527
f‐DBS	50.86 ± 12.13	50.50 ± 11.91	0.096	48.50 ± 11.63	0.014	50.50 ± 11.45	0.406	51.14 ± 11.40	0.968	52.93 ± 11.28	0.003
CDR	3.00 ± 0.00	3.00 ± 0.00	NA	3.00 ± 0.00	NA	3.00 ± 0.00	NA	3.00 ± 0.00	NA	3.00 ± 0.00	NA
NBM‐DBS	3.00 ± 0.00	3.00 ± 0.00	NA	3.00 ± 0.00	NA	3.00 ± 0.00	NA	3.00 ± 0.00	NA	3.00 ± 0.00	NA
f‐DBS	3.00 ± 0.00	3.00 ± 0.00	NA	3.00 ± 0.00	NA	3.00 ± 0.00	NA	3.00 ± 0.00	NA	3.00 ± 0.00	NA
Quality‐of‐life assessment											
BI	60.75 ± 17.94	59.75 ± 17.95	0.104	65.25 ± 17.73	0.004	62.25 ± 18.17	0.343	58.50 ± 17.63	0.119	57.00 ± 17.20	0.018
NBM‐DBS	59.17 ± 15.94	58.33 ± 17.51	0.611	62.50 ± 18.10	0.175	59.17 ± 17.72	1.000	55.83 ± 15.94	0.286	52.50 ± 14.75	0.102
f‐DBS	61.43 ± 19.26	60.36 ± 18.76	0.082	66.43 ± 18.13	0.013	63.57 ± 18.85	0.234	59.64 ± 18.76	0.292	58.93 ± 18.31	0.110
FAQ	27.00 ± 2.55	27.20 ± 2.71	0.248	26.20 ± 2.78	0.003	27.15 ± 2.64	0.546	27.60 ± 2.28	0.144	28.20 ± 2.09	0.003
NBM‐DBS	27.83 ± 1.33	27.83 ± 1.60	1.000	26.67 ± 1.63	0.013	27.67 ± 1.97	0.833	28.17 ± 1.94	0.679	28.33 ± 2.07	0.363
f‐DBS	26.64 ± 2.90	26.93 ± 3.08	0.206	26.00 ± 3.19	0.057	26.93 ± 2.92	0.435	27.36 ± 2.44	0.163	28.14 ± 2.18	0.004
FIM	71.50 ± 22.65	72.25 ± 23.11	0.170	77.00 ± 24.68	0.003	75.75 ± 24.52	0.011	70.20 ± 24.86	0.872	66.40 ± 22.41	0.023
NBM‐DBS	68.00 ± 20.07	69.00 ± 22.95	0.567	74.67 ± 28.54	0.165	72.67 ± 26.67	0.411	67.33 ± 30.66	0.911	63.50 ± 26.44	0.320
f‐DBS	73.00 ± 24.22	73.64 ± 23.90	0.332	78.00 ± 23.94	0.003	77.07 ± 24.47	0.023	71.43 ± 23.16	0.550	67.64 ± 21.44	0.021
Psychiatric symptoms assessment											
NPI	26.90 ± 4.17	26.75 ± 3.48	0.545	20.10 ± 4.04	0.000	20.40 ± 3.95	0.000	21.05 ± 3.86	0.000	21.55 ± 4.01	0.000
NBM‐DBS	28.83 ± 4.49	28.33 ± 3.50	0.415	16.33 ± 1.75	0.001	17.33 ± 2.73	0.003	18.00 ± 2.37	0.003	18.67 ± 2.66	0.003
f‐DBS	26.07 ± 3.89	26.07 ± 3.36	1.000	21.71 ± 3.65	0.000	21.71 ± 3.71	0.000	22.36 ± 3.67	0.000	22.79 ± 3.91	0.000
HAMA	16.30 ± 5.35	16.50 ± 5.24	0.403	12.95 ± 4.31	0.000	13.85 ± 4.46	0.000	14.10 ± 4.59	0.001	14.60 ± 4.57	0.002
NBM‐DBS	16.83 ± 5.23	17.67 ± 5.72	0.141	12.67 ± 4.27	0.006	14.00 ± 5.10	0.034	14.00 ± 5.37	0.129	15.33 ± 5.16	0.328
f‐DBS	16.07 ± 5.58	16.00 ± 5.16	0.828	13.07 ± 4.48	0.000	13.79 ± 4.37	0.000	14.14 ± 4.44	0.001	14.29 ± 4.46	0.000
HAMD	20.70 ± 5.35	20.50 ± 5.74	1.000	16.15 ± 4.76	0.000	16.85 ± 4.87	0.000	17.10 ± 4.63	0.000	17.55 ± 4.89	0.000
NBM‐DBS	21.00 ± 4.86	19.50 ± 5.58	0.248	14.83 ± 4.71	0.027	15.33 ± 4.37	0.028	15.50 ± 4.32	0.011	15.83 ± 5.04	0.066
f‐DBS	20.57 ± 5.72	20.93 ± 5.97	0.096	16.71 ± 4.84	0.001	17.50 ± 5.08	0.001	17.79 ± 4.74	0.001	18.29 ± 4.83	0.002
PSQI	14.40 ± 1.43	14.45 ± 1.32	0.739	10.85 ± 2.39	0.000	11.20 ± 1.91	0.000	11.35 ± 1.87	0.000	11.70 ± 1.75	0.000
NBM‐DBS	15.00 ± 1.10	15.33 ± 0.82	0.157	9.83 ± 3.87	0.028	10.33 ± 2.80	0.015	10.50 ± 2.81	0.020	10.67 ± 2.58	0.010
f‐DBS	14.14 ± 1.51	14.07 ± 1.33	0.705	11.29 ± 1.38	0.001	11.57 ± 1.34	0.001	11.71 ± 1.27	0.003	12.14 ± 1.10	0.003
Caregiver burden											
ZBI	50.55 ± 10.40	50.05 ± 10.92	0.106	45.20 ± 11.28	0.000	46.35 ± 10.52	0.000	46.65 ± 10.32	0.000	47.80 ± 9.72	0.014
NBM‐DBS	51.67 ± 6.74	50.17 ± 7.78	0.076	44.33 ± 9.07	0.018	45.33 ± 9.05	0.035	45.17 ± 8.28	0.023	46.00 ± 9.19	0.043
f‐DBS	50.07 ± 11.82	49.50 ± 11.70	0.775	45.57 ± 12.41	0.000	46.79 ± 11.39	0.002	47.29 ± 11.31	0.004	48.57 ± 10.17	0.159

**TABLE 4:** Baseline information and follow‐up data for all outcome measures.

Scales	Baseline	1 month	3 month	6 month	9 month	12 month	*p* (fvsNBM)
Cognitive assessments							
MMSE							0.993
NBM‐DBS	5.00 ± 2.19	4.50 ± 2.59	8.00 ± 3.85	6.17 ± 4.26	5.50 ± 4.04	4.33 ± 2.73	
f‐DBS	5.29 ± 3.41	5.43 ± 3.41	7.29 ± 4.16	5.86 ± 3.01	5.00 ± 3.01	4.57 ± 2.98	
MoCA							0.831
NBM‐DBS	4.33 ± 1.86	4.33 ± 1.75	7.00 ± 4.24	5.83 ± 4.02	4.33 ± 3.14	3.67 ± 2.50	
f‐DBS	4.21 ± 3.17	4.29 ± 2.95	5.64 ± 4.03	5.07 ± 3.34	4.43 ± 3.23	4.14 ± 2.96	
ADAS‐cog							0.891
NBM‐DBS	51.50 ± 10.50	51.33 ± 11.81	45.50 ± 16.36	49.00 ± 14.09	50.17 ± 12.42	52.33 ± 11.84	
f‐DBS	50.86 ± 12.13	50.50 ± 11.91	48.50 ± 11.63	50.50 ± 11.45	51.14 ± 11.40	52.93 ± 11.28	
CDR							NA
NBM‐DBS	3.00 ± 0.00	3.00 ± 0.00	3.00 ± 0.00	3.00 ± 0.00	3.00 ± 0.00	3.00 ± 0.00	—
f‐DBS	3.00 ± 0.00	3.00 ± 0.00	3.00 ± 0.00	3.00 ± 0.00	3.00 ± 0.00	3.00 ± 0.00	
Quality‐of‐life assessment							
BI							0.666
NBM‐DBS	59.17 ± 15.94	58.33 ± 17.51	62.50 ± 18.10	59.17 ± 17.72	55.83 ± 15.94	52.50 ± 14.75	
f‐DBS	61.43 ± 19.26	60.36 ± 18.76	66.43 ± 18.13	63.57 ± 18.85	59.64 ± 18.76	58.93 ± 18.31	
FAQ							0.397
NBM‐DBS	27.83 ± 1.33	27.83 ± 1.60	26.67 ± 1.63	27.67 ± 1.97	28.17 ± 1.94	28.33 ± 2.07	
f‐DBS	26.64 ± 2.90	26.93 ± 3.08	26.00 ± 3.19	26.93 ± 2.92	27.36 ± 2.44	28.14 ± 2.18	
FIM							0.704
NBM‐DBS	68.00 ± 20.07	69.00 ± 22.95	74.67 ± 28.54	72.67 ± 26.67	67.33 ± 30.66	63.50 ± 26.44	
f‐DBS	73.00 ± 24.22	73.64 ± 23.90	78.00 ± 23.94	77.07 ± 24.47	71.43 ± 23.16	67.64 ± 21.44	
Psychiatric symptoms assessment							
NPI							0.187
NBM‐DBS	28.83 ± 4.49	28.33 ± 3.50	16.33 ± 1.75	17.33 ± 2.73	18.00 ± 2.37	18.67 ± 2.66	
f‐DBS	26.07 ± 3.89	26.07 ± 3.36	21.71 ± 3.65	21.71 ± 3.71	22.36 ± 3.67	22.79 ± 3.91	
HAMA							0.824
NBM‐DBS	16.83 ± 5.23	17.67 ± 5.72	12.67 ± 4.27	14.00 ± 5.10	14.00 ± 5.37	15.33 ± 5.16	
f‐DBS	16.07 ± 5.58	16.00 ± 5.16	13.07 ± 4.48	13.79 ± 4.37	14.14 ± 4.44	14.29 ± 4.46	
HAMD							0.429
NBM‐DBS	21.00 ± 4.86	19.50 ± 5.58	14.83 ± 4.71	15.33 ± 4.37	15.50 ± 4.32	15.83 ± 5.04	
f‐DBS	20.57 ± 5.72	20.93 ± 5.97	16.71 ± 4.84	17.50 ± 5.08	17.79 ± 4.74	18.29 ± 4.83	
PSQI							0.480
NBM‐DBS	15.00 ± 1.10	15.33 ± 0.82	9.83 ± 3.87	10.33 ± 2.80	10.50 ± 2.81	10.67 ± 2.58	
f‐DBS	14.14 ± 1.51	14.07 ± 1.33	11.29 ± 1.68	11.57 ± 1.34	11.71 ± 1.27	12.14 ± 1.10	
Caregiver burden							
ZBI							0.859
NBM‐DBS	51.67 ± 6.74	50.17 ± 7.78	44.33 ± 9.07	45.33 ± 9.05	45.17 ± 8.28	46.00 ± 9.19	
f‐DBS	50.07 ± 11.82	50.05 ± 10.92	45.57 ± 12.41	46.79 ± 11.39	47.29 ± 11.31	48.57 ± 10.17	

**TABLE 5:** Statistics of the score improvement rate in both groups.

Scales	Follow‐up	NBM‐DBS	f‐DBS	Mean (95% CI)	*p*
Cognitive assessments					
MMSE	1 month	−0.50 ± 0.84	0.14 ± 0.86	0.64 (−0.24, 1.52)	0.135
	3 month	3.00 ± 2.61	1.57 ± 1.09	−1.00 (−2.93, 0.93)	0.120
	6 month	1.17 ± 3.43	0.57 ± 1.02	−0.60 (−4.19, 3.00)	0.330
	9 month	0.50 ± 3.39	−0.29 ± 0.83	−0.79 (−4.43, 2.77)	0.348
	12 month	−0.67 ± 2.42	−0.71 ± 0.83	−0.05 (−2.58, 2.49)	0.964
MoCA	1 month	0.00 ± 1.10	0.07 ± 0.62	0.07 (−1.08, 1.22)	0.894
	3 month	2.67 ± 2.94	1.43 ± 1.09	−1.24 (−3.09, 0.61)	0.145
	6 month	1.50 ± 2.81	0.86 ± 0.53	−0.64 (−3.59, 2.30)	0.389
	9 month	0.00 ± 1.79	0.21 ± 0.58	0.21 (−1.66, 2.09)	0.858
	12 month	−0.67 ± 1.51	−0.07 ± 1.83	0.60 (−0.49, 1.68)	0.337
ADAS‐cog	1 month	−0.17 ± 1.33	−0.36 ± 0.74	−0.19 (−1.58, 1.20)	0.664
	3 month	−6.00 ± 6.63	−2.36 ± 2.59	3.64 (−3.30, 10.58)	0.554
	6 month	−2.50 ± 4.59	−0.36 ± 2.56	2.14 (−1.19, 5.48)	0.194
	9 month	−1.33 ± 3.44	0.29 ± 2.76	1.62 (−1.42, 4.66)	0.278
	12 month	0.83 ± 3.37	2.07 ± 2.09	1.24 (−1.34, 3.82)	0.834
CDR	1 month	0	0	NA	NA
	3 month	0	0	NA	NA
	6 month	0	0	NA	NA
	9 month	0	0	NA	NA
	12 month	0	0	NA	NA
Quality‐of‐life assessment					
BI	1 month	−0.83 ± 3.76	−1.07 ± 2.13	−0.24 (−2.99, 2.51)	0.959
	3 month	3.33 ± 5.16	5.00 ± 6.50	1.67 (−4.65, 7.98)	0.535
	6 month	0.00 ± 8.37	2.14 ± 6.42	2.14 (−5.05, 9.33)	0.539
	9 month	−3.33 ± 6.83	−1.79 ± 6.08	1.55 (−4.91, 8.00)	0.621
	12 month	−6.67 ± 8.16	−2.50 ± 5.46	4.17 (−2.32, 10.65)	0.164
FAQ	1 month	0.00 ± 0.63	0.29 ± 0.83	0.29 (−0.51, 1.08)	0.375
	3 month	−1.17 ± 0.75	−0.64 ± 1.15	0.52 (−0.56, 1.61)	0.323
	6 month	−0.17 ± 1.83	0.29 ± 1.33	0.45 (−1.07, 1.97)	0.540
	9 month	0.33 ± 1.86	0.71 ± 1.86	0.38 (−1.52, 2.29)	0.865
	12 month	0.50 ± 1.22	1.50 ± 1.83	1.00 (−0.73, 2.73)	0.199
FIM	1 month	1.00 ± 4.00	0.64 ± 2.17	−0.36 (−3.23, 2.51)	0.797
	3 month	6.67 ± 10.05	5.00 ± 4.42	−1.67 (−12.18, 8.85)	0.619
	6 month	4.67 ± 12.74	4.07 ± 4.68	−0.60 (−8.60, 7.41)	0.051
	9 month	−0.67 ± 13.95	−1.57 ± 5.00	−0.90 (−15.50, 13.69)	0.264
	12 month	−4.50 ± 9.99	−5.36 ± 7.10	−0.86 (−9.07, 7.35)	0.455
Psychiatric symptoms assessment					
NPI	1 month	−0.50 ± 1.38	0.00 ± 0.96	0.50 (−0.62, 1.62)	0.543
	3 month	−12.50 ± 4.76	−4.36 ± 1.08	8.14 (3.15, 13.13)	0.002
	6 month	−11.50 ± 5.17	−4.36 ± 1.50	7.14 (1.73, 12.55)	0.019
	9 month	−10.83 ± 4.79	−3.71 ± 1.44	7.12 (2.10, 12.13)	0.014
	12 month	−10.17 ± 4.71	−3.29 ± 1.44	6.88 (1.95, 11.81)	0.015
HAMA	1 month	0.83 ± 1.17	−0.07 ± 1.21	−0.90 (−2.13, 0.32)	0.131
	3 month	−4.17 ± 2.23	−3.00 ± 1.57	1.17 (−0.65, 2.99)	0.195
	6 month	−2.83 ± 2.40	−2.29 ± 1.68	0.55 (−1.41, 2.51)	0.564
	9 month	−2.83 ± 3.82	−1.93 ± 1.59	0.90 (−3.09, 4.90)	0.596
	12 month	−1.50 ± 3.39	−1.79 ± 1.42	−0.29 (−3.83, 3.26)	0.849
HAMD	1 month	−1.50 ± 2.81	0.36 ± 0.74	1.86 (−1.09, 4.80)	0.101
	3 month	−6.17 ± 3.60	−3.86 ± 1.23	2.31 (−1.46, 6.08)	0.180
	6 month	−5.67 ± 3.83	−3.07 ± 1.21	2.60 (−1.41, 6.60)	0.160
	9 month	−5.50 ± 3.39	−2.79 ± 1.19	2.71 (−0.83, 6.26)	0.069
	12 month	−5.17 ± 4.58	−2.29 ± 1.33	2.88 (−1.91, 7.67)	0.187
PSQI	1 month	0.33 ± 0.52	−0.07 ± 0.73	−0.40 (−1.10, 0.29)	0.234
	3 month	−5.17 ± 4.12	−2.86 ± 1.29	2.31 (−2.00, 6.62)	0.232
	6 month	−4.67 ± 3.14	−2.57 ± 1.34	2.10 (−1.19, 5.38)	0.168
	9 month	−4.50 ± 3.27	−2.43 ± 1.55	2.07 (−1.35, 5.49)	0.295
	12 month	−4.33 ± 2.66	−2.00 ± 1.57	2.33 (−0.46, 5.12)	0.088
Caregiver burden					
ZBI	1 month	−1.50 ± 1.64	−0.07 ± 0.92	1.43 (−0.29, 3.15)	0.050
	3 month	−7.33 ± 5.20	−4.50 ± 2.95	2.83 (−0.98, 6.64)	0.136
	6 month	−6.33 ± 5.43	−3.29 ± 3.07	3.05 (−0.92, 7.02)	0.124
	9 month	−6.50 ± 4.89	−2.79 ± 2.99	3.71 (0.00, 7.43)	0.050
	12 month	−5.67 ± 5.16	−1.50 ± 3.76	4.17 (−1.30, 9.63)	0.057

### 3.3 Effects on Quality of Life

Baseline BI scores indicated significant daily living impairment but improved significantly at 3 months (65.25 ± 17.73, *p* < 0.05). FAQ scores showed a similar trend, peaking at 3 months (26.20 ± 2.78, *p* < 0.05). Notably, ZBI scores reflected a significant reduction in caregiver burden. The BI, FAQ, FIM, and ZBI scale scores at each time point were compared between the NBM‐DBS group and the fornix‐DBS group, with none indicating significant differences (*p* > 0.05). Bilateral NBM‐DBS and bilateral fornix‐DBS can enhance the quality of life and caregiver burden in patients with severe AD without significant differences (*p* > 0.05) (Figure 5, Tables 3, 4, 5).

### 3.4 Effects on Neuropsychiatric Symptoms and Sleep Disorders

At baseline, patients exhibited obvious neuropsychiatric symptoms (mean NPI score: 26.90 ± 4.17). In the third month post‐surgery, the NPI scores gradually decreased (20.10 ± 4.04 points, *p* < 0.05). Similarly, HAMA scores, reflecting anxiety, significantly improved 3 months postsurgery (12.95 ± 4.31 points, *p* < 0.05). HAMD scores, reflecting depression, showed significant early improvement (16.15 ± 4.76 points, *p* < 0.05). PSQI scores, measuring sleep quality, significantly improved 3 months postsurgery and maintained a downward trend (*p* < 0.05). NBM‐DBS demonstrated improvement in NPI scores compared to fornix‐DBS (*p* < 0.05) (Figure 5, Tables 3, 4, 5).

### 3.5 Study Adverse Events (AEs) and Long‐Term Prognosis

Table 6 showed 11 surgery‐related AEs in six patients (30.0%) in this study. Five patients (25%) in the fornix‐DBS group reported eight AEs, while only one (5%) in the NBM‐DBS group reported 3 AEs. In brief, four patients (20%) experienced mild symptoms like fatigue, depression, nausea, vomiting, falls, and IPG inflammation. They were effectively treated without serious consequences. Moreover, two patients (10.0%) in the fornix group developed serious AEs (subdural hematoma) but recovered well after timely reoperations. Six patients in the fornix group died during the follow‐up period. Among these deceased patients, four succumbed to severe pneumonia caused by COVID‐19 infection after unsuccessful treatment. One patient went missing 6 years postsurgery and died due to an accident. Another patient died from a stroke due to poor control of underlying conditions such as hypertension and diabetes.

**TABLE 6:** Summary of adverse events by category and treatment group.

Patient Characteristic	Value	f‐DBS	NBM‐DBS	*p*
Number	20	14	6	
Follow‐up time (months)	59.70 ± 28.41	75.36 ± 15.94	23.17 ± 10.85	0.00
Operation duration (hours)	5.92 ± 1.82	6.14 ± 2.08	5.42 ± 0.97	0.43
Length of hospital stay (days)	30.70 ± 16.69	29.64 ± 13.02	33.17 ± 24.63	0.804
Number of rehospitalizations (times)	1.40 ± 0.60	1.50 ± 0.65	1.17 ± 0.41	0.257
The number of dead	6	6	0	
The number of AE	6 (30%)	5 (25%)	1 (5%)	0.61
Number of AEs (person)	11 (55%)	8 (40%)	3 (15%)	0.77
Fatigue	1 (5%)	0	1 (5%)	
Depressed mood	1 (5%)	0	1 (5%)	
Naupathia	2 (10%)	1 (5%)	1 (5%)	
Emesia	1 (5%)	1 (5%)	0	
Fall	1 (5%)	1 (5%)	0	
Inflammation at the IPG site	1 (5%)	1 (5%)	0	
Postoperative pain	1 (5%)	1 (5%)	0	
Swelling and redness	1 (5%)	1 (5%)	0	
Reoperation of subdural hematoma (SAE)	2 (10%)	2 (10%)	0	

## 4 Discussion

We enrolled 20 patients with severe AD to assess the safety and long‐term efficacy of DBS on various symptom clusters. Our findings indicate that all participants exhibited good tolerance to DBS therapy, with initial improvements in cognitive function and overall quality of life. Although additional cognitive enhancements plateaued over time, there was a sustained reduction in neuropsychiatric symptoms and sleep disturbances, significantly enhancing patients' quality of life and reducing caregiver burden. Furthermore, our comparison of DBS targeting the NBM and the fornix revealed that NBM‐DBS demonstrated superior efficacy in managing neuropsychiatric symptoms. Both surgeries are well‐tolerated and safe for severe AD patients during the perioperative period. It is noteworthy that six patients in the fornix group died during the follow‐up period, possibly due to the progressive nature of AD and the longer postoperative duration in this group, which led to a more severe condition and increased mortality. Although the number of deaths in the fornix‐DBS group was higher, further follow‐up indicated that these deaths were mainly related to poor control of underlying diseases or concurrent infectious diseases rather than complications from the surgery itself. These findings emphasize that for AD, in addition to DBS, it is necessary to comprehensively manage underlying diseases and prevent complications to improve patients' overall survival rates and quality of life. Future research should further explore the comprehensive management of AD patients, including controlling underlying diseases, infection prevention, and postoperative care, to improve patients' long‐term prognosis.

Our research is the first to directly compare the fornix and the NBM as targets for DBS in severe AD, providing a systematic evaluation of their impact. The NBM‐DBS, which stimulates a hub of cholinergic neurons critical for cognition, attention, and memory, effectively alleviates neuropsychiatric symptoms by boosting cholinergic neurotransmission. In contrast, fornix‐DBS, which targets memory circuitry, has a more limited effect on these symptoms as it does not directly regulate the cholinergic system. Our analysis underscores the importance of selecting DBS targets based on their neural pathways and understanding the distinct roles of different brain regions in AD, particularly their connections to neuropsychiatric symptoms. By targeting the NBM, we hypothesize that DBS can modulate emotional and behavioral responses through its extensive interactions with the limbic system and prefrontal cortex, offering broader symptomatic relief. The hypothesis we propose is derived from a comprehensive analysis and review of existing professional literature, intended to provide a possible framework for explanation. However, the data in the article support the improvement of symptoms through DBS; the specific details of its mechanisms still require elucidation through future studies [35, 36, 37, 38, 39, 40]. This approach not only facilitates better communication between patients and families but also decreases reliance on psychotropic drugs, mitigates polypharmacy side effects, and elevates the quality of life for both patients and caregivers. Furthermore, it alleviates the burden on caregivers and improves patients' neuropsychiatric symptoms and sleep disorders. Our findings advocate for an approach to DBS in AD, emphasizing the importance of target selection and the potential for symptom relief beyond traditional pharmacological interventions, offering a treatment strategy for AD [41, 42, 43, 44].

In our study, a distinct trend was that patients with severe AD experienced an improvement in cognitive function within the first 3 months following DBS treatment. This initial improvement is likely due to the temporary alleviation of cognitive decline. However, the inherent irreversibility of AD's pathological process—characterized by β‐amyloid deposition, neurofibrillary tangles, and neuronal death [45, 46]—prevents a halt in disease progression. Moreover, the long‐term application of DBS may lead to neuroadaptive changes or stimulation tolerance, which can complicate the maintenance of therapeutic efficacy. Despite the promising short‐term benefits, the long‐term efficacy for cognitive function and quality of life in AD patients is constrained. DBS can modulate neural activity on a short‐term basis but is limited by its inability to reverse the extensive damage to neural circuits and neuronal loss that has already occurred [47]. The limited treatment capacity of DBS is also a factor, as it struggles to sustain long‐term symptomatic improvements in patients with severely compromised neural circuits, even after initial positive outcomes [48, 49, 50, 51, 52]. The uncertainty surrounding the optimal stimulation targets for DBS in AD further adds to the complexity of treatment outcomes, with variability observed across studies and the potential influence of individual differences and disease progression rates on the durability of therapeutic effects [53, 54, 55, 56]. The progression of AD to more advanced stages, even with temporary alleviation of symptoms, exacerbates the medical emergency and societal strain, increasing the demands on caregivers and the economic burden. It highlights the need for a more comprehensive understanding of DBS's role in AD treatment.

Prior research has indicated that DBS can improve cognitive function in patients with mild‐to‐moderate AD but does not seem to decelerate or reverse the progression of the disease. Moreover, these studies often overlook the substantial impact of neuropsychiatric symptoms, sleep disorders, and the resulting decline in quality of life and psychological stress on patients and their families. Additionally, there is a scarcity of research on the treatment of severe AD, but with the intensification of an aging population, the proportion of severe cases will increase, and currently, there are no effective intervention methods. Our study shows that DBS can alleviate cognitive dysfunction in the early stages but cannot reverse the decline in the long term or improve patient prognosis, which is similar to the conclusions drawn by Zhang and others in previous research. On this basis, we have further discovered that in addition to the improvement in cognitive function, the neuropsychiatric symptoms, sleep disorders, and caregiver burden of patients have been relieved and improved in the long term, especially in patients treated with NBM‐DBS, which will be a new potential clinical application with prospects. Building on existing literature, we acknowledge the role of DBS in modulating dopamine release, its interactions with limbic circuits, and its association with neuropsychiatric symptoms in dementia [57, 58]. It has demonstrated the effectiveness of NBM‐DBS in improving neuropsychiatric symptoms in Parkinson's disease (PD) and Lewy body dementia [59].

Given the pressing need for effective treatments in severe AD and the limitations of current therapeutic approaches, the exploration of DBS as a potential intervention is not just desirable but imperative. When traditional medications are insufficient, DBS can effectively mitigate symptoms like hallucinations, anxiety, and depression, improving the quality of life and simplifying caregiving. The unique advantages of DBS—such as its precision in targeting neural nuclei, reversibility, and minimal invasiveness—make it an attractive candidate for treating the cognitive decline associated with severe AD. By activating cholinergic neurons in the NBM, DBS could reduce amyloid‐beta levels and enhance cholinergic transmission, which is crucial for cognitive function [60, 61, 62]. Additionally, DBS may stimulate the release of neurotrophic factors, promoting dendritic growth and neurogenesis, and thus improving memory. The potential of DBS to inhibit neuroinflammation by reducing glial cell reactivity and subsequent neuronal loss is another significant area of interest [63]. Furthermore, DBS‐induced theta‐reset oscillations could optimize memory encoding by modifying the hippocampal neuronal structure and electrical activity [64, 65]. Lastly, DBS may regulate amyloid‐beta metabolism and reduce tau protein deposition, offering additional therapeutic benefits. The potential to offer a treatment that is both effective and safe for elderly patients, who often have comorbidities that complicate medication use, underscores the urgency of this research [66]. Our findings highlight the need for ongoing research into sustainable and effective treatments that address both cognitive and noncognitive aspects of AD to enhance patients' quality of life and reduce the societal burden of the disease. We recommend integrating DBS into a comprehensive AD treatment plan that includes pharmacotherapy and rehabilitation to ensure tailored care for optimal outcomes. Therefore, investing in robust, large‐scale studies to elucidate DBS's efficacy and safety in AD is essential to transforming this promising therapeutic modality into a standard of care. Future studies should aim to elucidate the mechanisms of DBS, assess its long‐term efficacy and safety, and explore personalized treatment strategies to maximize its therapeutic potential for AD patients, ultimately aiming to improve their symptoms and overall quality of life.

The study's limitations include a small sample size and nonrandomized design, impacting statistical power and generalizability. Firstly, the sample size is still relatively small, especially in the NBM‐DBS group. We will expand the sample size by adopting a broader recruitment strategy, including promoting multicenter cooperation to improve statistical power. Secondly, given the communication difficulties of AD patients, our primary data collection heavily relied on patients' families and caregivers. This approach may not fully reflect the patient's condition and severity of illness. For better understanding and optimizing treatment, future studies should delve deeper into DBS mechanisms and explore more objective assessment methods. It includes integrating multisource data like patient self‐assessment, nursing observations, biomarkers, and imaging to create a comprehensive health and treatment evaluation system. Advanced technologies like wearable devices and smartphones can enhance data accuracy and depth. While our study design considered multiple outcome measures, resource constraints limited their full realization. However, we recognize their importance and plan to enhance our framework in future studies. Thirdly, this study highlighted the clinical outcomes of DBS but was limited in exploring its underlying mechanisms. Future research should delve into its detailed molecular and neurotransmitters and dynamically monitor post‐DBS neural loop and brain network changes using advanced imaging and electrophysiological techniques. Lastly, this study is a nonrandomized controlled study. We deeply analyzed the potential bias introduced by patient preferences when selecting the DBS treatment target (fornix or NBM), including selection bias, performance bias, and evaluation bias. To mitigate these biases, we adopted multiple strategies: adjusting baseline data through statistical analysis to reduce selection bias and using third‐party management for data collection and analysis to minimize performance and evaluation bias. Despite the challenges, we are committed to improving the science and credibility of research through these measures, aiming to provide a more solid and reliable evidence base for the clinical treatment of AD.

## 5 Conclusion

DBS is a potential therapeutic approach for severe AD, capable of improving patients' cognitive function, quality of life, and neuropsychiatric symptoms. Notably, NBM‐DBS showed distinct advantages in ameliorating neuropsychiatric symptoms, providing valuable insights for clinically selecting the optimal DBS target. Future studies should assess long‐term efficacy and safety, personalize DBS treatments based on genetics, brain scans, and clinical presentations, and explore DBS's synergy with drug therapy, cognitive training, and other approaches.

## Author Contributions

Junpeng Xu, Bin Liu, and Zhebin Feng were responsible for conducting the formal analysis, contributing to the methodology, overseeing the project, and writing the original draft of the manuscript. Guosong Shang and Zhebin Feng were involved in curating the data and collecting the necessary resources. Haonan Yang and Yuhan Chen were involved in the conception of the study, as well as writing and reviewing the manuscript. Xinguang Yu and Zhiqi Mao played a supervisory role in the study and contributed to writing, reviewing, and editing the manuscript. The article was a collaborative effort by all authors, who approved the final version of the submitted manuscript.

## Disclosure

The authors declare that this research was conducted in an objective and unbiased manner. No commercial or financial interests related to the outcomes of the study have been declared by the authors. All authors have contributed significantly to the research and preparation of the manuscript, and all data have been accurately represented. The study was funded by (STI 2030—Major Projects [2021ZD0200407]), and the funders had no role in the study design, data collection, analysis, or interpretation. The corresponding author had full access to all the data in the study and had final responsibility for the decision to submit for publication. The study complies with all ethical regulations, and approval was obtained from the relevant institutional review board. Informed consent was received from all participants involved in the study. Data underlying the study findings are available upon request without undue reservation.

## Ethics Statement

The trial has been approved by the Medical Ethics Committee of the Chinese PLA General Hospital (S2015‐013‐02).

## Conflicts of Interest

The authors declare no conflicts of interest.

## Supporting information


Data S1.

Data S2.

## Data Availability

The dataset and material are available from the corresponding author upon reasonable request.
