# Supplementary figures and images for: Efficacy and Safety of Bilateral Deep Brain Stimulation (DBS) for Severe Alzheimer's Disease: A Comparative Analysis of Fornix Versus Basal Ganglia of Meynert
Source: CNS Neurosci Ther. 2025 Apr 17;31(4):e70285. doi: 10.1111/cns.70285 (PMC12004396; doi:10.1111/cns.70285)

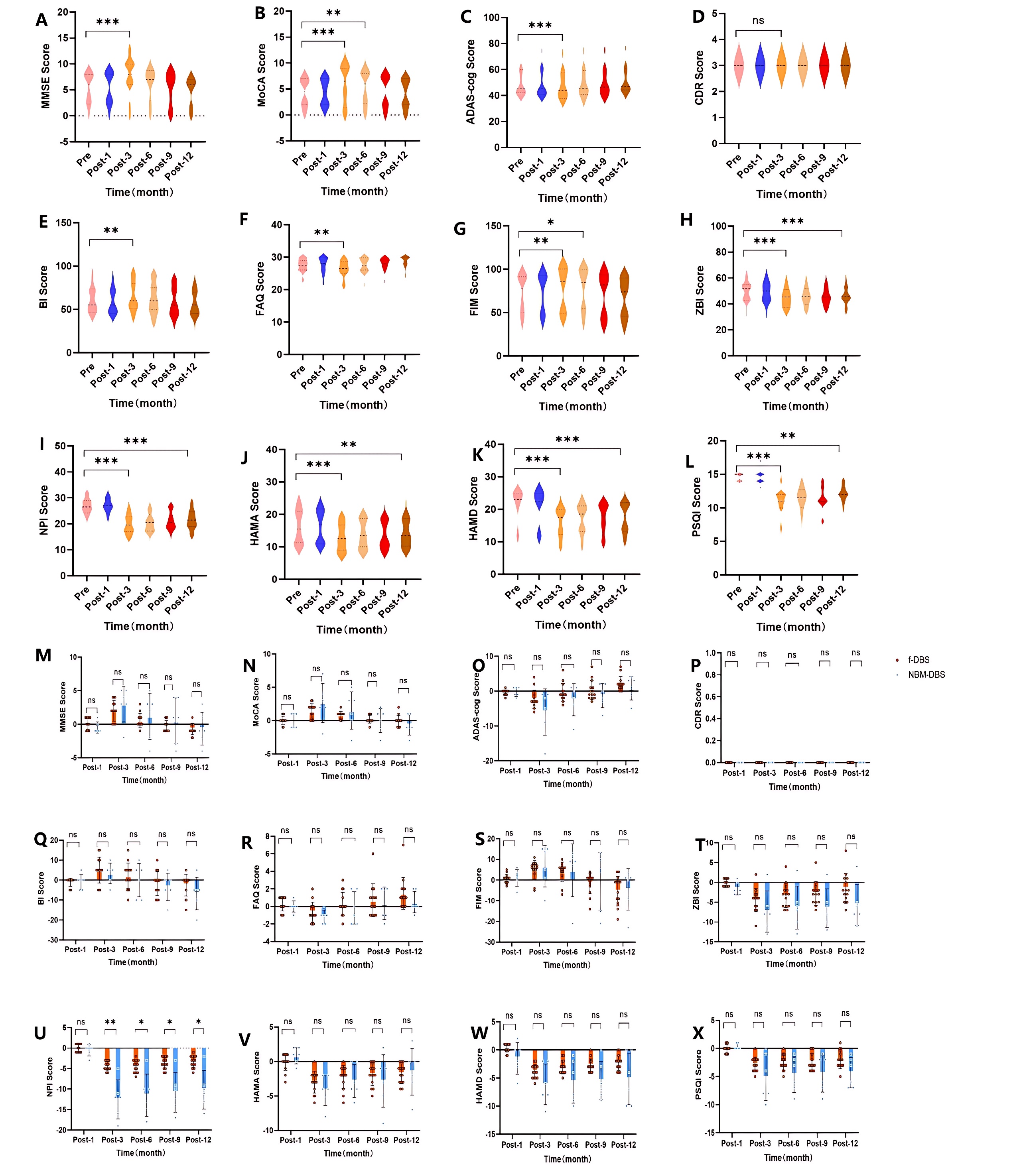

Supplement: Supplementary file 1 — Data S1. [file CNS-31-e70285-s002.jpg]

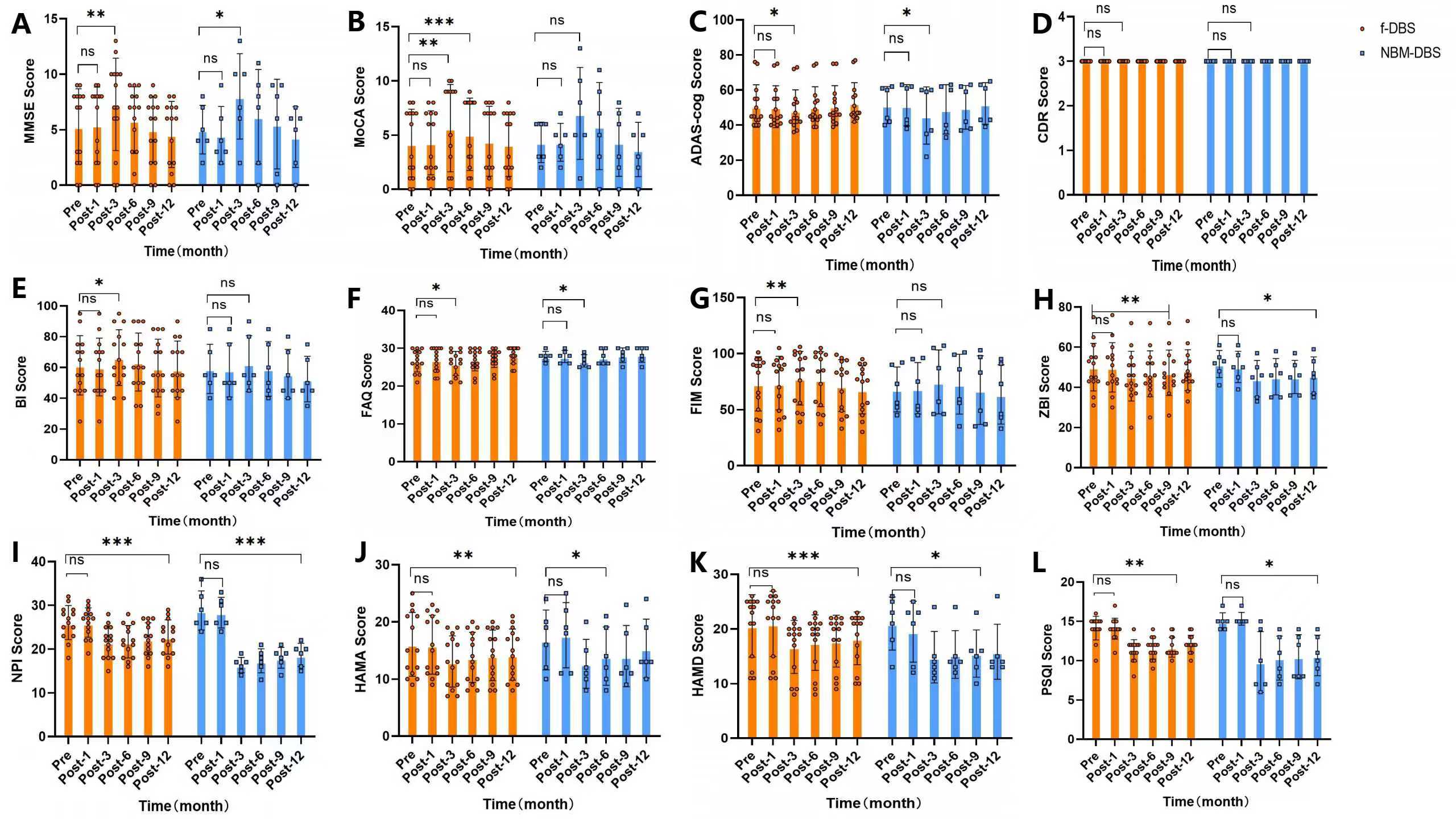

Supplement: Supplementary file 2 — Data S2. [file CNS-31-e70285-s001.jpg]
